# Mechanically Gated Ion Channels in Mammalian Hair Cells

**DOI:** 10.3389/fncel.2018.00100

**Published:** 2018-04-11

**Authors:** Xufeng Qiu, Ulrich Müller

**Affiliations:** ^1^Solomon H. Snyder Department of Neuroscience, Johns Hopkins University School of Medicine, Baltimore, MD, United States; ^2^Department of Cell Biology, Johns Hopkins University School of Medicine, Baltimore, MD, United States

**Keywords:** hair cell, inner ear, mechanotransduction, auditory, LHFPL5, TMIE, TMC1, PIEZO2

## Abstract

Hair cells in the inner ear convert mechanical stimuli provided by sound waves and head movements into electrical signal. Several mechanically evoked ionic currents with different properties have been recorded in hair cells. The search for the proteins that form the underlying ion channels is still in progress. The mechanoelectrical transduction (MET) channel near the tips of stereociliary in hair cells, which is responsible for sensory transduction, has been studied most extensively. Several components of the sensory mechanotransduction machinery in stereocilia have been identified, including the multi-transmembrane proteins tetraspan membrane protein in hair cell stereocilia (TMHS)/LHFPL5, transmembrane inner ear (TMIE) and transmembrane channel-like proteins 1 and 2 (TMC1/2). However, there remains considerable uncertainty regarding the molecules that form the channel pore. In addition to the sensory MET channel, hair cells express the mechanically gated ion channel PIEZO2, which is localized near the base of stereocilia and not essential for sensory transduction. The function of PIEZO2 in hair cells is not entirely clear but it might have a role in damage sensing and repair processes. Additional stretch-activated channels of unknown molecular identity and function have been found to localize at the basolateral membrane of hair cells. Here, we review current knowledge regarding the different mechanically gated ion channels in hair cells and discuss open questions concerning their molecular composition and function.

## Introduction

**Figure 1:**
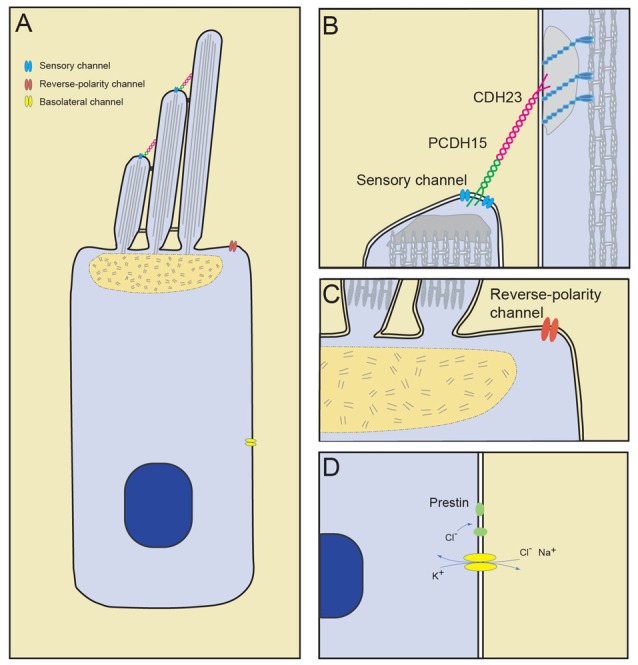
Mechanically gated ion channels in hair cells. **(A)** Diagram of a hair cell with the sensory channel located at the tips of the shorter stereocilia, reverse-polarity channel in the apical cell surface and stretch-activated channels in the basolateral membrane. **(B)** The sensory transduction channel is localized near the lower end of tip links, which consists of PCDH15 and CDH23. **(C)** The reverse-polarity channel is concentrated near the base of the longest stereocilia. **(D)** Basolateral currents carried by unknown channels. Cl^−^ influx through basolateral channel may drive motor protein prestin transitions.

**Figure 2:**
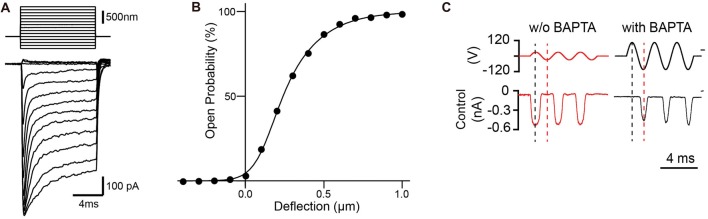
Mechanotransduction currents measured with stiff probe or fluid jets. **(A)** Representative transduction currents in outer hair cells (OHCs) in response to a set of 10 ms hair bundle deflections with a stiff probe ranging from −400 nm to 1000 nm with 100 nm steps. **(B)** Representative plot of open probability with hair bundle deflection from **(A)**, fitted with a three Boltzmann model. **(C)** Representative mechanotransduction currents in response to sinusoidal deflection of hair bundles at P5 for a wild-type C57BL/6 mouse with and without BAPTA treatment to break tip links. Stimulus monitor, the driving voltage to the fluid jet, is shown at the top. A positive driving voltage denotes displacement toward the tallest edge of the hair bundle. In controls the response after BAPTA treatment occurs in the opposite phase (reverse-polarity) of the stimulus compared to the response prior to BAPTA treatment.

Hair cells of the inner ear are specialized mechanosensory cells, which convert mechanical stimuli provided by sound waves (cochlea) or head movement (vestibular system) into electrical signals. Hair cells are highly polarized cells with extraordinary morphological specialization for sensing mechanical stimuli. The most prominent morphological specialization of a hair cell is the hair bundle. It protrudes from the apical surface of a hair cell and is formed by an array of F-actin based stereocilia that are arranged in a staircase of decreasing heights (Figure 1A; reviewed in Gillespie and Müller, 2009; Schwander et al., 2010). The sensory mechanoelectrical transduction (MET) channel in hair cells is localized near the tips of stereocilia at the base of the tip link filament that connects a shorter stereocilium to its next taller neighbor (Figures 1A,B; Pickles et al., 1984; Beurg et al., 2008). Deflection of the hair bundle towards the tallest stereocilia leads to an increase in the MET channel open probability, while deflections in the opposite direction decrease channel open probability (Figures 2A,B; Hudspeth and Corey, 1977; Ohmori, 1985; Crawford et al., 1989; Kros et al., 1992; Nicolson et al., 1998). Tip links thus connect stereocilia in the direction of their greatest mechanical sensitivity. Tip links have been proposed to transmit mechanical force onto the transduction channel and possibly to act as the gating spring that regulates channel function (Corey and Hudspeth, 1983). Consistent with this model, transduction is lost when tip links are disrupted (Assad et al., 1991) and direct pulling on the tip link opens the MET channel (Basu et al., 2016).

Besides the sensory MET channels at tip links, a second mechanically activated channel was recently identified in hair cells that is located at their apical cell surface where stereocilia emanate from the cell body (Figures 1A,C; Beurg et al., 2016; Wu et al., 2017). This MET channel was initially observed after disrupting the function of the sensory MET channel. It was described as a reverse-polarity MET channel since it was activated by deflections of the hair bundles towards the shortest stereocilia and thus opposite to the normal direction that activates the sensory MET channel (Alagramam et al., 2011; Kim et al., 2013; Beurg et al., 2014; Marcotti et al., 2014). Subsequent studies demonstrated that this channel is stretch-activated (Beurg et al., 2016); its function in hair cells is still under investigation as described below.

Hair cells in the mammalian cochlea come in two flavors, outer hair cells (OHCs) and inner hair cells (IHCs). OHCs have an important function in amplifying input sound signals while IHCs transmit sound information to the CNS (reviewed in Dallos, 2008; Kazmierczak and Müller, 2012; Safieddine et al., 2012). Notably, the basolateral membrane of OHCs is a highly specialized compartment that is thought to be important for the amplification of sound. OHCs show a phenomenon called electromotility where the length of the cell body is regulated by membrane potential. The hair cell shortens during hair cell depolarization and lengthens during hyperpolarization (Brownell et al., 1985; Kachar et al., 1986; Ashmore, 1987). The length of a hair cell is also affected by mechanical stimuli that are applied to the basolateral membrane (Brundin et al., 1989; Brundin and Russell, 1994). The motor protein prestin, which is localized in the basolateral membrane of OHCs but not IHCs, is critical for electromotility (Belyantseva et al., 2000; Zheng et al., 2000), but little is known about other proteins that might contribute to this process. The changes in the length of the cell body provides a mechanical signal, which could activate MET channels that in turn might affect the amplification process. Stretch activated currents carried by ion channels of unknown molecular identity have been observed at the basolateral surface of OHCs in guinea-pig (Figures 1A,D; Ding et al., 1991; Iwasa et al., 1991; Rybalchenko and Santos-Sacchi, 2003). These ionic currents and the underlying MET channels are least well studied and we know little about their function.

In the following, we will summarize current knowledge regarding the properties and molecular composition of the various mechanically gated ion channels in hair cells.

## Properties of Mechanically Gated Ion Channels in Hair Cells

### Sensory Transduction Channels

The activity of the sensory MET channel at the tips of stereocilia can be recorded in organotypic culture as an inward current following deflection of the hair bundle with a stiff probe (Figures 2A,B) or fluid jet (Figure 2C). Initial studies of hair cells from the bullfrog saccule showed that the MET channel opens within ~40 μs (Corey and Hudspeth, 1979b), but larger deflection gate the channel much more quickly (Corey and Hudspeth, 1983). In turtles, activation kinetics is also in the microseconds range and varies tonotopically (Ricci et al., 2005). Kinetics in mammalian cochlear hair cells is so fast that it has been difficult to determine accurately by conventional force probes (Ricci et al., 2005), but may be directly measured with new technology in the near future (Doll et al., 2012). The fast activation kinetics has led to the idea that the channel is directly gated by mechanical force without intervening second messengers (Corey and Hudspeth, 1983).

The MET channel is non-selective for cations (Corey and Hudspeth, 1979a; Kros et al., 1992; Farris et al., 2004) but has a higher selectivity for Ca^2+^ compared to other cations (Lumpkin et al., 1997; Ricci and Fettiplace, 1998; Beurg et al., 2006). In physiological condition, hair bundles are immersed in endolymph, which is high in K^+^ (154 mM) and low in Ca^2+^ (0.03 mM; Bosher and Warren, 1971, 1978). Most of the ionic current through the transduction channel is therefore carried by K^+^. However, Ca^2+^ profoundly affects channel function where channel activity is increased when the external Ca^2+^ is decreased from a mM to a μM concentration (Corey and Hudspeth, 1983; Ohmori, 1985; Ricci et al., 2003; Pan et al., 2012).

The organ of Corti in mammals has the ability to separate sound frequencies along its length—high-frequency tones at the proximal end and low-frequency at the distal end of the organ. The Ca^2+^ selectivity and single-channel conductance also show tonotopic characteristics in OHCs but not in IHCs. In 20 μM external Ca^2+^, single-channel conductance varies from 145 to 210 pS for OHCs along the tonotopic axis but is about 260 pS for IHCs along the entire length of the cochlea (Beurg et al., 2006, 2014, 2015b). Similar observations had previously been made in turtle (Ricci et al., 2003). It is currently not clear why a tonotopic gradient in conductance is observed only in OHCs but not in IHCs. It suggests that OHCs might have an active role in decoding mechanical signals at different frequencies that are then transmitted to IHCs. Perhaps this has to do with adaptation rates and frequency tuning. Changes in conductance will affect adaptation rates and thus the speed by which channels are able to respond to a new incoming stimulus.

Sensory MET channels in hair cells adapt to mechanical stimuli, which leads to a decrease in current during a constant stimulus but additional stimulation again increases current. Adaptation is thought to set the resting tension of the transduction channel to position the channel near the most sensitive point of activation, and is important for providing amplification for mechanical signals (reviewed in LeMasurier and Gillespie, 2005). Two components of transducer current adaptation, fast and slow, were observed in turtle, frog and mammalian hair cells (Figure 2A; Howard and Hudspeth, 1987; Crawford et al., 1991; Wu et al., 1999; Eatock, 2000; Holt and Corey, 2000). Fast adaptation has been proposed to be caused by binding of Ca^2+^ either to the MET channel itself or to a binding side near the channel. Slow adaptation is thought to be regulated by a myosin motor complex at the upper insertion site of tip links (Crawford et al., 1989, 1991; Choe et al., 1998; Cheung and Corey, 2006). However, there is still considerable debate regarding the mechanism of adaptation and its regulation by Ca^2+^ (Peng et al., 2013; Corns et al., 2014). One possibility is that adaptation varies among different species and different type of hair cells. Original studies of hair cells in the bullfrog saccule showed significant fast and slow adaptation (Corey and Hudspeth, 1983; Assad et al., 1989). Later studies in mammalian cochlear hair cells, which operate at much higher frequencies compared to vestibular hair cells, suggested that fast adaptation predominates in hair cells of the cochlea (Kennedy et al., 2003; Waguespack et al., 2007; Peng et al., 2013). Recent findings by the Ricci laboratory indicate that fast adaptation in cochlear hair cells is independent of both Ca^2+^ entry and voltage, while channel open probability is modulated by divalent ions interacting with the local lipid environment (Peng et al., 2013, 2016). However, others have concluded that adaption even in cochlear hair cells is dependent on Ca^2+^ influx (Corns et al., 2014). The discrepancies between the different studies might be explained by differences in the way hair bundles were stimulated. While Peng and colleagues used stiff probes for hair bundle stimulation, Corns and colleagues used fluid jets to deflect hair bundles. The importance of the lipid environment in regulating MET channels was highlighted by the role of PIP2 in hair bundles. Acute modulation of free PIP2 in stereocilia causes changes in channel properties, including loss of fast adaptation, increase resting open probability, reduction of single-channel conductance, and reduction of Ca^2+^ selectivity (Effertz et al., 2017). This is remarkable because PIP2 affects properties that were previously thought to be intrinsic to the channel. Other factors such as cyclic AMP may also contribute to channel activation and adaptation (Ricci and Fettiplace, 1997), suggesting multiple-pathways of regulation for MET.

### Reverse-Polarity Channels

During the early development of hair cells, their hair bundles are less directionally sensitive. Transducer currents can be observed by deflection of the hair bundle both towards the shortest and longest stereocilia (Waguespack et al., 2007; Kindt et al., 2012; Kim et al., 2013; Marcotti et al., 2014). This might in part be the case because stereocilia of developing hair bundles are less well organized and they are connected by an abundance of linkages between stereocilia such as ankle links, side links, tip links and top connectors; following hair cell maturation, only tip links and top connectors remain (Goodyear et al., 2003). In IHCs and OHCs of the cochlea, reverse-polarity currents are detectable at birth but decline subsequently in parallel to maturation of the normal polarity MET current (Beurg et al., 2016). However, the reverse-polarity currents are detectable even in more mature hair cells when MET is blocked by disrupting tip-links with BAPTA treatment (Marcotti et al., 2014; Wu et al., 2017; Figure 2C) or by gene mutations that affect components of the MET machinery (Stepanyan and Frolenkov, 2009; Alagramam et al., 2011; Kim et al., 2013; Zhao et al., 2014; Beurg et al., 2015b, 2016). Notably, earlier studies in isolated guinea pig hair cells already described a tip-link independent mechanotransduction current (Meyer et al., 1998, 2005). This inward current could be inhibited by statically deflecting hair bundles towards the shortest stereocilia (Meyer et al., 2005). Since reverse-polarity currents that are observed after tip-link breakage and after inactivation of the sensory MET channel increase in parallel to a decrease in normal polarity currents, it had been proposed that normal and reverse-polarity currents share a similar pore protein (Kim et al., 2013; Beurg et al., 2015b, 2016). Further studies showed that the channel properties such as ion permeability, conductance, regulation of conductance by Ca^2+^, and sensitivity to channel blockers are similar but not identical between the two channels (Beurg et al., 2014, 2016; Marcotti et al., 2014). Single-channel conductance for the reverse-polarity channel has been determined to be ~60 pS at 1.5 mM Ca^2+^ and ~90 pS at 0.07 mM Ca^2+^ (Beurg et al., 2014, 2016), which differs from the conductance of the normal polarity channel. Unlike the normal polarity currents in OHCs, the reverse-polarity currents also showed no tonotopic gradient in conductance. Adaptation kinetics for the reverse-polarity currents was much faster and more complete when compared to the normal-polarity currents (Beurg et al., 2014). Thus, the relationship between the two currents remained unclear for some time and has only recently been clarified by molecular studies (see below).

### Basolateral Currents

Several studies identified stretch activated MET currents in the basolateral membrane of hair cells, but the properties of these currents differed between reports. At least three different currents that are affected by mechanical force have been reported in OHCs. One type of current was activated by stretch and a single-channel conductance of 38–50 pS was determined for the underlying channel. This ion channel was non-selective to cations and had a reversal potential ~ −12 mV (Ding et al., 1991). The second current was also activated by stretch but a large conductance around 130–150 pS was reported. The ion selectivity of this second channel was not fully determined, but initial studies suggested that it was possibly selective for K^+^ and Na^+^ (Iwasa et al., 1991). Finally, a third type of stretch-sensitive conductance was observed that was non-selective for cations and anions. Based on its permeability for Cl^−^, which is thought to regulate prestin function (Oliver et al., 2001), a role in cochlea amplification was proposed for this third conductance (Figure 1D; Rybalchenko and Santos-Sacchi, 2003).

## Molecular Composition of Mechanotransdution Channels in Hair Cells

### Sensory Transduction Channels

**Figure 3:**
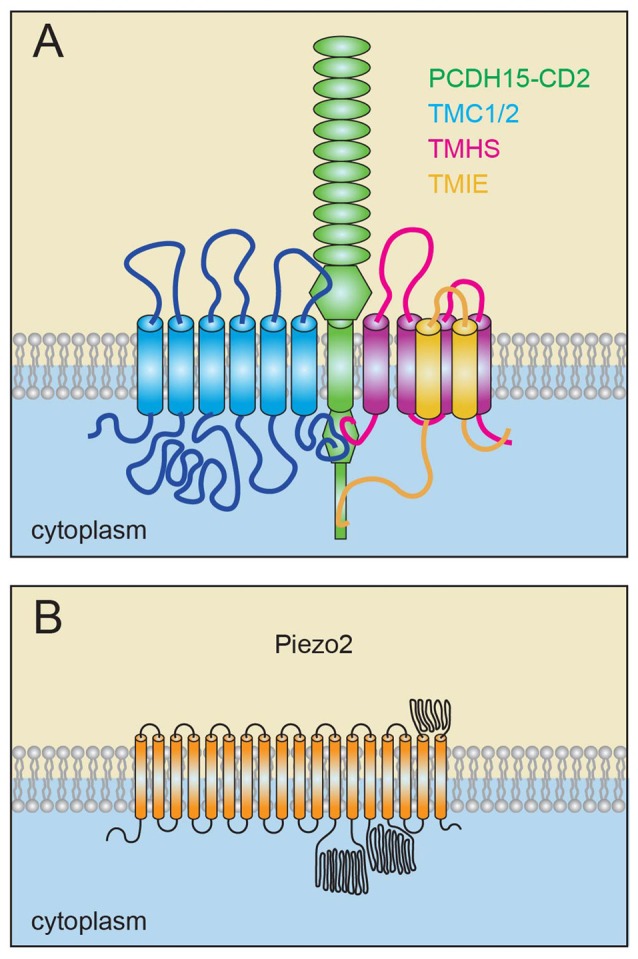
Model of the sensory transduction channel and for PIEZO2. **(A)** Transmembrane channel-like proteins 1 and 2 (TMC1/2), tetraspan membrane protein in hair cell stereocilia (TMHS)/LHFPL5 and transmembrane inner ear (TMIE) bind to PCDH15 and are constituents of the sensory mechanoelectrical transduction (MET) machinery. TMC1/2 and TMHS/LHFPL5 bind to PCDH15. TMIE binds to TMHS/LHFPL5 as well as to the unique C-terminal domain of one specific PCDH15 isoform in stereocilia. **(B)** Model of the PIEZO2 channel, which contain at least 18 transmembrane domains and potentially up to 38 transmembrane domains.

The search for the molecular constituents of the MET channel in stereocilia has been in progress for decades. Using high speed Ca^2+^ imaging, it was demonstrated that the sensory MET channel is localized near the lower end of tip links (Beurg et al., 2009). Subsequent studies that were driven by the analysis of mouse mutants carrying mutations in genes that cause deafness identified several multi-transmembrane proteins that are critical for MET and are also concentrated near the lower end of tip links. These are transmembrane channel-like proteins 1 and 2 (TMC1 and TMC2; Pan et al., 2013; Maeda et al., 2014; Kurima et al., 2015), tetraspan membrane protein in hair cell stereocilia (TMHS; also known as Lipoma HMGIC Fusion Partner-Like 5; LHFPL5; Xiong et al., 2012; Mahendrasingam et al., 2017), and transmembrane inner ear expressed gene (TMIE; Zhao et al., 2014; Figure 3A, Table 1). Other proteins such as CIB2 and TOMT are also essential for mechanotransduction (Riazuddin et al., 2012; Cunningham et al., 2017; Erickson et al., 2017; Giese et al., 2017; Michel et al., 2017; Wang et al., 2017) but will not be considered here because they do not encode proteins with multiple transmembrane domains and therefore are not predicted to be components contributing to the pore of the mechanotransduction channel. TMC1 and TMC2 have been proposed to be the pore-forming components of the mechanosensory channels (Corey and Holt, 2016), but direct evidence for this hypothesis is still lacking (Wu and Müller, 2016) and the role of LHFPL5 and TMIE in the channel complex still needs to be determined.

**Table 1:** Candidate components of the mechanotransduction channel in hair cells and how they affect channel activity.

Candidates	Stereocillia localization	MET requirement	Channel properties changed in mutant mice				Heterologous expression
			Rise time	Adaptation	Single-channel conductance	Calcium permeability	
TMC1/2	Yes	Required	N/A	Slower	Conflict	Altered	Intracellular
LHFPL5	Yes	Required	Slower	Slower	Reduced	N/D	Cell surface together with Pcdh15
TMIE	Yes	Required	N/A	N/A	N/A	N/A	Cell surface

*Tmc1* and *Tmc2* are members of a gene family consisting in mammals of eight genes (Keresztes et al., 2003; Kurima et al., 2003). *Tmc1* and *Tmc4* are the main family members that are expressed in adult cochlear hair cells, while *Tmc2* is only transiently expressed in the cochlea during early postnatal development but can be detected in vestibular hair cells into adulthood (Kawashima et al., 2011; Liu et al., 2014; Scheffer et al., 2015). Although *Tmc3* belongs to the same gene subfamily as *Tmc1* and *Tmc2*, it does not appear to be essential for MET by hair cells (Beurg et al., 2014). Cumulative studies during the past several years suggest that TMC1 and TMC2 are intimately associated with the MET channel in hair cells and candidates for pore-forming subunits for several reasons. First, mutations in the gene encoding TMC1 cause dominant and recessive forms of hearing loss in humans and mice (Kurima et al., 2002; Vreugde et al., 2002). Second, studies with genetically modified mice have shown that both TMC1 and TMC2 contribute to MET in cochlear hair cells at early postnatal ages and expression of either TMC1 or TMC2 can rescue MET in *Tmc1/2* deficient hair cells (Kawashima et al., 2011; Pan et al., 2013; Askew et al., 2015). Third, immunohistochemical studies with antibodies indicated that TMC1/2 proteins are localized to hair bundles. Similarly, epitope-tagged versions of TMC1/2 expressed in hair cells with the help of viruses or in BAC-transgenic mice are expressed in hair bundles and some of the protein is concentrated in the tip-link region (Askew et al., 2015; Kurima et al., 2015). Fourth, yeast two-hybrid screens and co-immunoprecipitation experiments provide evidence that TMC1/2 binds to PCDH15 (Maeda et al., 2014; Beurg et al., 2015b), which is a component of the tip-link in proximity to the transduction channel (Figure 1B; Ahmed et al., 2006; Kazmierczak et al., 2007). Finally, MET channel properties are affected by TMC1 and TMC2. Single-channel conductance, Ca^2+^ selectivity and adaptation time constant in developing hair cells lacking either TMC1 alone or TMC2 alone differ (Kim and Fettiplace, 2013; Pan et al., 2013; Corns et al., 2017). The tonotopic gradient in single-channel conductance normally observed in OHCs is diminished in hair cells lacking TMC1. Conversely, the Ca^2+^ selectivity of IHCs and OHCs lacking TMC2 but not TMC1 is significantly reduced (Kim and Fettiplace, 2013; Pan et al., 2013; Beurg et al., 2014). Finally, a missense mutation in *Tmc1* has been reported to reduce Ca^2+^ permeability and single-channel conductance in IHCs (Pan et al., 2013).

However, whether TMC1 and TMC2 form the channel pore is still under debate. It was proposed that the tonotopic gradient in the conductance and Ca^2+^ selectivity of the MET channel can be explained by variations in the stoichiometry of TMC1/2 (Pan et al., 2013). However, TMC2 is not expressed in adult hair cells, TMC1 and TMC2 show little co-localization in hair cells, and TMC2 mutations do not affect hearing function (Kawashima et al., 2011; Kurima et al., 2015). In addition, a second study could not confirm that a missense mutation in *Tmc1* reduces single-channel conductance (Beurg et al., 2015a) as initially reported (Pan et al., 2013). Surprisingly, a recent study has also shown that all changes in the properties of the MET current that have been reported for mice with mutations in *Tmc1* and *Tmc2* can be caused by modulating the concentration of PIP2 in hair bundles (Effertz et al., 2017), indicating that these changes are not necessarily directly linked to the channel pore. Finally, no mechanical sensing function for TMCs was found so far in invertebrates. A *tmc* ortholog in the worm has been reported to relate to sodium-sensitive channel for salt sensation (Chatzigeorgiou et al., 2013), but subsequent studies did not confirm this finding and suggested that the worm protein has instead a function in pH sensing (Wang et al., 2016). Others showed a sexual and metabolic function for TMC1 in *C. elegans* (Zhang et al., 2015) and a modulatory role of TMC1/2 for membrane excitability through a background leak conductance (Yue et al., 2018). In *Drosophila*, TMC was found to play a function in providing sensory feedback for laval locomotion (Guo et al., 2016). Additional study showed a role in food texture sensation for *Drosophila* TMC (Zhang et al., 2016). Critically, TMC proteins from mammals and invertebrates could so far not be expressed at the cell surface of heterologous cells, and appear to be largely retained in the ER (Labay et al., 2010; Zhao et al., 2014; Guo et al., 2016; Zhang et al., 2016). Thus, while TMC1/2 are plausible candidates to contribute to the channel pore of the MET channel, further studies are necessary to determine their role in the transduction complex (Table 1).

TMHS/LHFPL5 is a second protein that has been implicated to be an integral component of the mechanotransduction channel in hair cells. TMHS/LHFPL5 is a member of a small subfamily within the large superfamily of proteins with four transmembrane domains (Petit et al., 1999; Kalay et al., 2006). Immunohistochemical studies have shown that TMHS/LHFPL5 is localized in developing and adult hair cells near the lower end of tip-links where the transduction channel is localized (Xiong et al., 2012; Beurg et al., 2015b; Mahendrasingam et al., 2017). Biochemical experiments have shown that TMHS/LHFPL5 binds to the C-terminus of the tip-link protein PCDH15, but so far no interactions with TMC1/2 could be demonstrated (Beurg et al., 2015b). *Tmhs/Lhfpl5* mutations cause deafness and lead to a dramatic reduction in mechanotransduction currents in cochlear hair cells of mice (Xiong et al., 2012). Further studies demonstrated that TMHS/LHFPL5 regulates the transport of PCDH15 and TMC1 into the stereocilia of hair cells thus affecting the assembly of tip links and the transduction complex (Xiong et al., 2012; Beurg et al., 2015b). However, TMHS/LHFPL5 is not absolutely essential for protein transport and up to 30% of stereocilia still assemble tip link complexes in the absence of TMHS/LFHPL5 (Xiong et al., 2012). This has facilitated the study of the properties of the remaining transduction complexes in *Tmhs/Lhfpl5*-deficient hair cells. Single channel recordings demonstrated that in the absence of TMHS/LHFPL5 the conductance of the MET channel is affected as well as its activation and adaptation kinetics (Table 1, Xiong et al., 2012). The tonotopic gradient that is normally observed in the conductance of the MET channel in OHCs is also dramatically diminished in the mutants (Beurg et al., 2015b). Taken together, the findings suggest that TMHS/LHFPL5 is an integral component of MET complex (Xiong et al., 2012; Beurg et al., 2015b). Since some residual current remains in hair cells lacking TMHS/LHFPL5, it is unlikely that this protein alone forms the pore of the MET channel. One possibility is that TMHS/LHFPL5 is an auxiliary subunit of the pore-forming subunits of the transduction channel much like TARP proteins are for AMPA receptors; TARP proteins share structural similarity to TMHS/LHFPL5 and regulate the transport and conductance properties of AMPA receptors (Xiong et al., 2012). However, TMHS/LHFPL5 could also be part of a heteromeric channel and contribute to the channel pore.

TMIE is a protein with two transmembrane domains and linked to deafness in both human and mice (Mitchem et al., 2002; Naz et al., 2002). TMIE was identified as a binding partner of PCDH15 and TMHS using yeast two-hybrid screens (Zhao et al., 2014). Interactions with TMC1/2 could so far not be demonstrated (Zhao et al., 2014). Further studies demonstrated that TMIE is localized to the tips of the stereocilia near the transduction machinery and binds to a splice variant of PCDH15 (PCDH15-CD2) that is directly implicated in regulating MET (Zhao et al., 2014). Strikingly, in *Tmie*-deficient cochlear hair cells, no MET currents can be detected, even though tip links remain intact and all known components of the MET machinery including TMC1/2 can travel into stereocilia (Zhao et al., 2014). Overexpress the C-terminal fragment of TMIE, which contain the binding domain mediating interactions with PCDH15 and TMHS/LHFPL5 disrupt transduction. Similarly, transduction is disrupted by expression of a PCDH15 protein fragment that perturbs interactions between PCDH15 and TMIE. Taken together, these findings suggest that PCDH15, TMIE and TMHS/LHFPL5 form a ternary complex critical for MET (Zhao et al., 2014; Figure 3A). Nevertheless, the precise function of TMIE in the transduction complex remain to be established. Heterologously expressed TMIE has so far not been shown to form an ion channel (Table 1, Zhao et al., 2014), but TMIE is a candidate protein to be integral to the transduction channel and possibly contributing to its pore.

### Reverse-Polarity Channels

The similarities in single-channel conductance and pharmacological properties of the normal and reverse-polarity current in hair cells initially suggest that these two mechanically gated currents are carried by the same channel pore (Kim et al., 2013; Beurg et al., 2014, 2015b, 2016). However, others noted significant differences between normal and reverse-polarity currents (Marcotti et al., 2014). Intriguingly, the reverse-polarity current shares characteristics with currents carried by mechanically gated ion channels PIEZO1 and PIEZO2. PIEZO1 and PIEZO2 were identified in a functional screen as bona fide MET channels in mammals (Coste et al., 2010, 2012). They are by far the largest ion channels identified and contain a large number of transmembrane domains (Figure 3B). Similar to the reverse-polarity currents in hair cells, currents carried by PIEZO1 and PIEZO2 are rapidly activated by mechanical force and adapt much faster than typical MET currents in hair cell stereocilia (Coste et al., 2012; Beurg et al., 2014). Wu et al. (2017) subsequently demonstrated that PIEZO2 but not PIEZO1 is expressed in mechanosensory hair cells. However, the function of the sensory MET channel is normal in PIEZO2 mutant mice. Instead, reverse-polarity currents are completely abolished in mice lacking PIEZO2 function in hair cells (Wu et al., 2017). Immunohistochemical studies demonstrated that PIEZO2 is localized at the apical surface of hair cells near the base of stererocilia with highest concentration near the tallest stereocilia (Wu et al., 2017). The function of PIEZO2 in mechanosensory hair cells is still unclear. PIEZO2 activity is observed after the sensory MET machinery in stereocilia is disrupted, suggesting regulatory crosstalk between the two MET channels that appears to be regulated by the intracellular Ca^2+^ concentration (Wu et al., 2017). Interestingly, the assembly of the sensory MET complex in stereocilia during development appears to be affected in the absence of PIEZO2 and hearing function is slightly affected in adult mutants (Wu et al., 2017). It is therefore tempting to speculate that PIEZO2 has an important repair function in hair cell, similar to its role in bone (Ivanusic, 2017).

### Basolateral Currents

We still know next to nothing about the molecular composition of ion channels that carry the stretch activated currents in the basolateral membrane of OHCs (Ding et al., 1991; Iwasa et al., 1991; Rybalchenko and Santos-Sacchi, 2003). Since OHCs undergo length-changes during mechanical amplification, it is tempting to speculate that MET channels in OHCs are in some way related to the amplification processes. As noted above, the prestin protein that located in the basolateral membrane of OHCs but not IHCs is critical for sound amplification (Belyantseva et al., 2000; Zheng et al., 2000). Notably, Cl^−^ influx through a stretch-sensitive channel in the basolateral membrane of OHCs was reported to allosteric modulate prestin, thus potentially functioning in OHC amplification (Oliver et al., 2001; Rybalchenko and Santos-Sacchi, 2003). A candidate protein to be stretch activated in OHCs is PIEZO2. Although it is concentrated in the apical surface of hair cells (Wu et al., 2017), it cannot be excluded that it is also present at lower levels in the basolateral membrane. However, the ion-selectivity of PIEZO2 does not fit with a role in passing anions and studies with *Piezo2*-deficient hair cells demonstrated that PIEZO2 is not essential for electromotility (Wu et al., 2017). Thus, further studies are necessary to identify the proteins that form the stretch-activated ion channels in the basolateral membrane of hair cells and to determine their function.

## Conclusion

Recent studies have provided compelling evidence that hair cells express several molecularly distinct ion channels with different function. The best studied of these is the sensory MET channel at tips of stereocilia. Substantial evidence suggests that TMC1/2, TMHS and TMIE are integral components of the sensory MET channel (Figure 3A) but which protein(s) form the channel pore remains to be established. Far less is known about the molecular composition and function of stretch activated ion channels in the cell body of hair cells. PIEZO2 has recently been shown to be an integral component of the stretch-activated MET channel in the apical surface of hair cells (Figure 3B), but virtually nothing is known about the molecular composition of the stretch-activated MET channels located in the basolateral surface of hair cells. The function of the MET channels in the cell bodies of hair cells also remains to be established. Intriguing questions are how the different ion channels are targeted to different compartments in hair cells and the extent to which they engage in regulatory crosstalk.

## Author Contributions

XQ and UM wrote the manuscript. Figures were designed by XQ.

## Conflict of Interest Statement

UM is a co-founder of Decibel Therapeutics. The other author declares that the research was conducted in the absence of any commercial or financial relationships that could be construed as a potential conflict of interest.

## References

[B1] Ahmed Z. M. Goodyear R. Riazuddin S. Lagziel A. Legan P. K. Behra M.. (2006). The tip-link antigen, a protein associated with the transduction complex of sensory hair cells, is protocadherin-15. J. Neurosci. 26, 7022–7034. 10.1523/JNEUROSCI.1163-06.2006 16807332 PMC6673907

[B2] Alagramam K. N. Goodyear R. J. Geng R. Furness D. N. van Aken A. F. Marcotti W.. (2011). Mutations in protocadherin 15 and cadherin 23 affect tip links and mechanotransduction in mammalian sensory hair cells. PLoS One 6:e19183. 10.1371/journal.pone.0019183 21532990 PMC3080917

[B3] Ashmore J. F. (1987). A fast motile response in guinea-pig outer hair cells: the cellular basis of the cochlear amplifier. J. Physiol. 388, 323–347. 10.1113/jphysiol.1987.sp016617 3656195 PMC1192551

[B4] Askew C. Rochat C. Pan B. Asai Y. Ahmed H. Child E.. (2015). Tmc gene therapy restores auditory function in deaf mice. Sci. Transl. Med. 7:295ra108. 10.1126/scitranslmed.aab1996 26157030 PMC7298700

[B5] Assad J. A. Hacohen N. Corey D. P. (1989). Voltage dependence of adaptation and active bundle movement in bullfrog saccular hair cells. Proc. Natl. Acad. Sci. U S A 86, 2918–2922. 10.1073/pnas.86.8.2918 2468161 PMC287031

[B6] Assad J. A. Shepherd G. M. Corey D. P. (1991). Tip-link integrity and mechanical transduction in vertebrate hair cells. Neuron 7, 985–994. 10.1016/0896-6273(91)90343-x 1764247

[B7] Basu A. Lagier S. Vologodskaia M. Fabella B. A. Hudspeth A. J. (2016). Direct mechanical stimulation of tip links in hair cells through DNA tethers. Elife 5:e16041. 10.7554/eLife.16041 27331611 PMC4951189

[B8] Belyantseva I. A. Adler H. J. Curi R. Frolenkov G. I. Kachar B. (2000). Expression and localization of prestin and the sugar transporter GLUT-5 during development of electromotility in cochlear outer hair cells. J. Neurosci. 20:RC116. 11125015 10.1523/JNEUROSCI.20-24-j0002.2000 PMC6773019

[B9] Beurg M. Evans M. G. Hackney C. M. Fettiplace R. (2006). A large-conductance calcium-selective mechanotransducer channel in mammalian cochlear hair cells. J. Neurosci. 26, 10992–11000. 10.1523/JNEUROSCI.2188-06.2006 17065441 PMC6674673

[B10] Beurg M. Fettiplace R. Nam J. H. Ricci A. J. (2009). Localization of inner hair cell mechanotransducer channels using high-speed calcium imaging. Nat. Neurosci. 12, 553–558. 10.1038/nn.2295 19330002 PMC2712647

[B11] Beurg M. Goldring A. C. Fettiplace R. (2015a). The effects of Tmc1 Beethoven mutation on mechanotransducer channel function in cochlear hair cells. J. Gen. Physiol. 146, 233–243. 10.1085/jgp.201511458 26324676 PMC4555472

[B15] Beurg M. Xiong W. Zhao B. Müller U. Fettiplace R. (2015b). Subunit determination of the conductance of hair-cell mechanotransducer channels. Proc. Natl. Acad. Sci. U S A 112, 1589–1594. 10.1073/pnas.1420906112 25550511 PMC4321290

[B12] Beurg M. Goldring A. C. Ricci A. J. Fettiplace R. (2016). Development and localization of reverse-polarity mechanotransducer channels in cochlear hair cells. Proc. Natl. Acad. Sci. U S A 113, 6767–6772. 10.1073/pnas.1601067113 27162344 PMC4914197

[B13] Beurg M. Kim K. X. Fettiplace R. (2014). Conductance and block of hair-cell mechanotransducer channels in transmembrane channel-like protein mutants. J. Gen. Physiol. 144, 55–69. 10.1085/jgp.201411173 24981230 PMC4076520

[B14] Beurg M. Nam J. H. Crawford A. Fettiplace R. (2008). The actions of calcium on hair bundle mechanics in mammalian cochlear hair cells. Biophys. J. 94, 2639–2653. 10.1529/biophysj.107.123257 18178649 PMC2267152

[B16] Bosher S. K. Warren R. L. (1971). A study of the electrochemistry and osmotic relationships of the cochlear fluids in the neonatal rat at the time of the development of the endocochlear potential. J. Physiol. 212, 739–761. 10.1113/jphysiol.1971.sp009354 5557069 PMC1395717

[B17] Bosher S. K. Warren R. L. (1978). Very low calcium content of cochlear endolymph, an extracellular fluid. Nature 273, 377–378. 10.1038/273377a0 661948

[B18] Brownell W. E. Bader C. R. Bertrand D. de Ribaupierre Y. (1985). Evoked mechanical responses of isolated cochlear outer hair cells. Science 227, 194–196. 10.1126/science.3966153 3966153

[B20] Brundin L. Flock A. Canlon B. (1989). Sound-induced motility of isolated cochlear outer hair cells is frequency-specific. Nature 342, 814–816. 10.1038/342814a0 2601740

[B19] Brundin L. Russell I. (1994). Tuned phasic and tonic motile responses of isolated outer hair cells to direct mechanical stimulation of the cell body. Hear. Res. 73, 35–45. 10.1016/0378-5955(94)90280-1 8157504

[B21] Chatzigeorgiou M. Bang S. Hwang S. W. Schafer W. R. (2013). tmc-1 encodes a sodium-sensitive channel required for salt chemosensation in *C. elegans*. Nature 494, 95–99. 10.1038/nature11845 23364694 PMC4021456

[B22] Cheung E. L. Corey D. P. (2006). Ca^2+^ changes the force sensitivity of the hair-cell transduction channel. Biophys. J. 90, 124–139. 10.1142/9789812773456_0047 16214875 PMC1367012

[B23] Choe Y. Magnasco M. O. Hudspeth A. J. (1998). A model for amplification of hair-bundle motion by cyclical binding of Ca^2+^ to mechanoelectrical-transduction channels. Proc. Natl. Acad. Sci. U S A 95, 15321–15326. 10.1073/pnas.95.26.15321 9860967 PMC28041

[B24] Corey D. P. Holt J. R. (2016). Are TMCs the mechanotransduction channels of vertebrate hair cells? J. Neurosci. 36, 10921–10926. 10.1523/JNEUROSCI.1148-16.2016 27798174 PMC5098833

[B25] Corey D. P. Hudspeth A. J. (1979a). Ionic basis of the receptor potential in a vertebrate hair cell. Nature 281, 675–677. 10.1038/281675a0 45121

[B26] Corey D. P. Hudspeth A. J. (1979b). Response latency of vertebrate hair cells. Biophys. J. 26, 499–506. 10.1016/s0006-3495(79)85267-4 318064 PMC1328566

[B27] Corey D. P. Hudspeth A. J. (1983). Kinetics of the receptor current in bullfrog saccular hair cells. J. Neurosci. 3, 962–976. 6601694 10.1523/JNEUROSCI.03-05-00962.1983 PMC6564517

[B28] Corns L. F. Jeng J. Y. Richardson G. P. Kros C. J. Marcotti W. (2017). TMC2 modifies permeation properties of the mechanoelectrical transducer channel in early postnatal mouse cochlear outer hair cells. Front. Mol. Neurosci. 10:326. 10.3389/fnmol.2017.00326 29093662 PMC5651230

[B29] Corns L. F. Johnson S. L. Kros C. J. Marcotti W. (2014). Calcium entry into stereocilia drives adaptation of the mechanoelectrical transducer current of mammalian cochlear hair cells. Proc. Natl. Acad. Sci. U S A 111, 14918–14923. 10.1073/pnas.1409920111 25228765 PMC4205606

[B30] Coste B. Mathur J. Schmidt M. Earley T. J. Ranade S. Petrus M. J.. (2010). Piezo1 and Piezo2 are essential components of distinct mechanically activated cation channels. Science 330, 55–60. 10.1126/science.1193270 20813920 PMC3062430

[B31] Coste B. Xiao B. Santos J. S. Syeda R. Grandl J. Spencer K. S.. (2012). Piezo proteins are pore-forming subunits of mechanically activated channels. Nature 483, 176–181. 10.1038/nature10812 22343900 PMC3297710

[B32] Crawford A. C. Evans M. G. Fettiplace R. (1989). Activation and adaptation of transducer currents in turtle hair cells. J. Physiol. 419, 405–434. 10.1113/jphysiol.1989.sp017878 2621635 PMC1190013

[B33] Crawford A. C. Evans M. G. Fettiplace R. (1991). The actions of calcium on the mechano-electrical transducer current of turtle hair cells. J. Physiol. 434, 369–398. 10.1113/jphysiol.1991.sp018475 1708822 PMC1181423

[B34] Cunningham C. L. Wu Z. Jafari A. Zhao B. Schrode K. Harkins-Perry S.. (2017). The murine catecholamine methyltransferase mTOMT is essential for mechanotransduction by cochlear hair cells. Elife 6:e33307. 10.7554/eLife.33307 28504928 PMC5462538

[B35] Dallos P. (2008). Cochlear amplification, outer hair cells and prestin. Curr. Opin. Neurobiol. 18, 370–376. 10.1016/j.conb.2008.08.016 18809494 PMC2630119

[B36] Ding J. P. Salvi R. J. Sachs F. (1991). Stretch-activated ion channels in guinea pig outer hair cells. Hear. Res. 56, 19–28. 10.1016/0378-5955(91)90149-4 1722801

[B37] Doll J. C. Peng A. W. Ricci A. J. Pruitt B. L. (2012). Faster than the speed of hearing: nanomechanical force probes enable the electromechanical observation of cochlear hair cells. Nano Lett. 12, 6107–6111. 10.1021/nl3036349 23181721 PMC3549426

[B38] Eatock R. A. (2000). Adaptation in hair cells. Annu. Rev. Neurosci. 23, 285–314. 10.1146/annurev.neuro.23.1.285 10845066

[B39] Effertz T. Becker L. Peng A. W. Ricci A. J. (2017). Phosphoinositol-4,5-bisphosphate regulates auditory hair-cell mechanotransduction-channel pore properties and fast adaptation. J. Neurosci. 37, 11632–11646. 10.1523/JNEUROSCI.1351-17.2017 29066559 PMC5707765

[B40] Erickson T. Morgan C. P. Olt J. Hardy K. Busch-Nentwich E. Maeda R.. (2017). Integration of Tmc1/2 into the mechanotransduction complex in zebrafish hair cells is regulated by Transmembrane O-methyltransferase (Tomt). Elife 6:e1993. 10.7759/cureus.1993 28534737 PMC5462536

[B41] Farris H. E. LeBlanc C. L. Goswami J. Ricci A. J. (2004). Probing the pore of the auditory hair cell mechanotransducer channel in turtle. J. Physiol. 558, 769–792. 10.1113/jphysiol.2004.061267 15181168 PMC1665030

[B42] Giese A. P. J. Tang Y. Q. Sinha G. P. Bowl M. R. Goldring A. C. Parker A.. (2017). CIB2 interacts with TMC1 and TMC2 and is essential for mechanotransduction in auditory hair cells. Nat. Commun. 8:43. 10.1038/s41467-017-00061-1 28663585 PMC5491523

[B43] Gillespie P. G. Müller U. (2009). Mechanotransduction by hair cells: models, molecules, and mechanisms. Cell 139, 33–44. 10.1016/j.cell.2009.09.010 19804752 PMC2888516

[B44] Goodyear R. J. Legan P. K. Wright M. B. Marcotti W. Oganesian A. Coats S. A.. (2003). A receptor-like inositol lipid phosphatase is required for the maturation of developing cochlear hair bundles. J. Neurosci. 23, 9208–9219. 14534255 10.1523/JNEUROSCI.23-27-09208.2003 PMC6740823

[B45] Guo Y. Wang Y. Zhang W. Meltzer S. Zanini D. Yu Y.. (2016). Transmembrane channel-like (tmc) gene regulates *Drosophila* larval locomotion. Proc. Natl. Acad. Sci. U S A 113, 7243–7248. 10.1073/pnas.1606537113 27298354 PMC4932980

[B46] Holt J. R. Corey D. P. (2000). Two mechanisms for transducer adaptation in vertebrate hair cells. Proc. Natl. Acad. Sci. U S A 97, 11730–11735. 10.1073/pnas.97.22.11730 11050202 PMC34342

[B47] Howard J. Hudspeth A. J. (1987). Mechanical relaxation of the hair bundle mediates adaptation in mechanoelectrical transduction by the bullfrog’s saccular hair cell. Proc. Natl. Acad. Sci. U S A 84, 3064–3068. 10.1073/pnas.84.9.3064 3495007 PMC304803

[B48] Hudspeth A. J. Corey D. P. (1977). Sensitivity, polarity and conductance change in the response of vertebrate hair cells to controlled mechanical stimuli. Proc. Natl. Acad. Sci. U S A 74, 2407–2411. 10.1073/pnas.74.6.2407 329282 PMC432181

[B49] Ivanusic J. J. (2017). Molecular mechanisms that contribute to bone marrow pain. Front. Neurol. 8:458. 10.3389/fneur.2017.00458 28955292 PMC5601959

[B50] Iwasa K. H. Li M. X. Jia M. Kachar B. (1991). Stretch sensitivity of the lateral wall of the auditory outer hair cell from the guinea pig. Neurosci. Lett. 133, 171–174. 10.1016/0304-3940(91)90562-8 1726184

[B51] Kachar B. Brownell W. E. Altschuler R. Fex J. (1986). Electrokinetic shape changes of cochlear outer hair cells. Nature 322, 365–368. 10.1038/322365a0 3736662

[B52] Kalay E. Li Y. Uzumcu A. Uyguner O. Collin R. W. Caylan R.. (2006). Mutations in the lipoma HMGIC fusion partner-like 5 (LHFPL5) gene cause autosomal recessive nonsyndromic hearing loss. Hum. Mutat. 27, 633–639. 10.1002/humu.20368 16752389

[B53] Kawashima Y. Géléoc G. S. Kurima K. Labay V. Lelli A. Asai Y.. (2011). Mechanotransduction in mouse inner ear hair cells requires transmembrane channel-like genes. J. Clin. Invest. 121, 4796–4809. 10.1172/JCI60405 22105175 PMC3223072

[B54] Kazmierczak P. Müller U. (2012). Sensing sound: molecules that orchestrate mechanotransduction by hair cells. Trends Neurosci. 35, 220–229. 10.1016/j.tins.2011.10.007 22177415 PMC3310959

[B55] Kazmierczak P. Sakaguchi H. Tokita J. Wilson-Kubalek E. M. Milligan R. A. Muller U.. (2007). Cadherin 23 and protocadherin 15 interact to form tip-link filaments in sensory hair cells. Nature 449, 87–91. 10.1038/nature06091 17805295

[B56] Kennedy H. J. Evans M. G. Crawford A. C. Fettiplace R. (2003). Fast adaptation of mechanoelectrical transducer channels in mammalian cochlear hair cells. Nat. Neurosci. 6, 832–836. 10.1038/nn1089 12872124

[B57] Keresztes G. Mutai H. Heller S. (2003). TMC and EVER genes belong to a larger novel family, the TMC gene family encoding transmembrane proteins. BMC Genomics 4:24. 10.1186/1471-2164-4-24 12812529 PMC165604

[B59] Kim K. X. Beurg M. Hackney C. M. Furness D. N. Mahendrasingam S. Fettiplace R. (2013). The role of transmembrane channel-like proteins in the operation of hair cell mechanotransducer channels. J. Gen. Physiol. 142, 493–505. 10.1085/jgp.201311068 24127526 PMC3813385

[B58] Kim K. X. Fettiplace R. (2013). Developmental changes in the cochlear hair cell mechanotransducer channel and their regulation by transmembrane channel-like proteins. J. Gen. Physiol. 141, 141–148. 10.1085/jgp.201210913 23277480 PMC3536526

[B60] Kindt K. S. Finch G. Nicolson T. (2012). Kinocilia mediate mechanosensitivity in developing zebrafish hair cells. Dev. Cell 23, 329–341. 10.1016/j.devcel.2012.05.022 22898777 PMC3426295

[B61] Kros C. J. Rüsch A. Richardson G. P. (1992). Mechano-electrical transducer currents in hair cells of the cultured neonatal mouse cochlea. Proc. Biol. Sci. 249, 185–193. 10.1098/rspb.1992.0102 1280836

[B63] Kurima K. Ebrahim S. Pan B. Sedlacek M. Sengupta P. Millis B. A.. (2015). TMC1 and TMC2 Localize at the site of mechanotransduction in mammalian inner ear hair cell stereocilia. Cell Rep. 12, 1606–1617. 10.1016/j.celrep.2015.07.058 26321635 PMC4569002

[B64] Kurima K. Peters L. M. Yang Y. Riazuddin S. Ahmed Z. M. Naz S.. (2002). Dominant and recessive deafness caused by mutations of a novel gene, TMC1, required for cochlear hair-cell function. Nat. Genet. 30, 277–284. 10.1038/ng842 11850618

[B65] Kurima K. Yang Y. Sorber K. Griffith A. J. (2003). Characterization of the transmembrane channel-like (TMC) gene family: functional clues from hearing loss and epidermodysplasia verruciformis. Genomics 82, 300–308. 10.1016/s0888-7543(03)00154-x 12906855

[B66] Labay V. Weichert R. M. Makishima T. Griffith A. J. (2010). Topology of transmembrane channel-like gene 1 protein. Biochemistry 49, 8592–8598. 10.1021/bi1004377 20672865 PMC3005947

[B67] LeMasurier M. Gillespie P. G. (2005). Hair-cell mechanotransduction and cochlear amplification. Neuron 48, 403–415. 10.1016/j.neuron.2005.10.017 16269359

[B68] Liu H. Pecka J. L. Zhang Q. Soukup G. A. Beisel K. W. He D. Z. (2014). Characterization of transcriptomes of cochlear inner and outer hair cells. J. Neurosci. 34, 11085–11095. 10.1523/JNEUROSCI.1690-14.2014 25122905 PMC4131018

[B69] Lumpkin E. A. Marquis R. E. Hudspeth A. J. (1997). The selectivity of the hair cell’s mechanoelectrical-transduction channel promotes Ca^2+^ flux at low Ca^2+^ concentrations. Proc. Natl. Acad. Sci. U S A 94, 10997–11002. 10.1073/pnas.94.20.10997 9380748 PMC23561

[B70] Maeda R. Kindt K. S. Mo W. Morgan C. P. Erickson T. Zhao H.. (2014). Tip-link protein protocadherin 15 interacts with transmembrane channel-like proteins TMC1 and TMC2. Proc. Natl. Acad. Sci. U S A 111, 12907–12912. 10.1073/pnas.1402152111 25114259 PMC4156717

[B71] Mahendrasingam S. Fettiplace R. Alagramam K. N. Cross E. Furness D. N. (2017). Spatiotemporal changes in the distribution of LHFPL5 in mice cochlear hair bundles during development and in the absence of PCDH15. PLoS One 12:e0185285. 10.1371/journal.pone.0185285 29069081 PMC5656302

[B72] Marcotti W. Corns L. F. Desmonds T. Kirkwood N. K. Richardson G. P. Kros C. J. (2014). Transduction without tip links in cochlear hair cells is mediated by ion channels with permeation properties distinct from those of the mechano-electrical transducer channel. J. Neurosci. 34, 5505–5514. 10.1523/JNEUROSCI.4086-13.2014 24741041 PMC3988408

[B73] Meyer J. Furness D. N. Zenner H. P. Hackney C. M. Gummer A. W. (1998). Evidence for opening of hair-cell transducer channels after tip-link loss. J. Neurosci. 18, 6748–6756. 9712646 10.1523/JNEUROSCI.18-17-06748.1998 PMC6792952

[B74] Meyer J. Preyer S. Hofmann S. I. Gummer A. W. (2005). Tonic mechanosensitivity of outer hair cells after loss of tip links. Hear. Res. 202, 97–113. 10.1016/j.heares.2004.11.013 15811703

[B75] Michel V. Booth K. T. Patni P. Cortese M. Azaiez H. Bahloul A.. (2017). CIB2, defective in isolated deafness, is key for auditory hair cell mechanotransduction and survival. EMBO Mol. Med. 9, 1711–1731. 10.15252/emmm.201708087 29084757 PMC5709726

[B76] Mitchem K. L. Hibbard E. Beyer L. A. Bosom K. Dootz G. A. Dolan D. F.. (2002). Mutation of the novel gene Tmie results in sensory cell defects in the inner ear of spinner, a mouse model of human hearing loss DFNB6. Hum. Mol. Genet. 11, 1887–1898. 10.1093/hmg/11.16.1887 12140191

[B77] Naz S. Giguere C. M. Kohrman D. C. Mitchem K. L. Riazuddin S. Morell R. J.. (2002). Mutations in a novel gene, TMIE, are associated with hearing loss linked to the DFNB6 locus. Am. J. Hum. Genet. 71, 632–636. 10.1086/342193 12145746 PMC379198

[B78] Nicolson T. Rusch A. Friedrich R. W. Granato M. Ruppersberg J. P. Nüsslein-Volhard C. (1998). Genetic analysis of vertebrate sensory hair cell mechanosensation: the zebrafish circler mutants. Neuron 20, 271–283. 10.1016/s0896-6273(00)80455-9 9491988

[B79] Ohmori H. (1985). Mechano-electrical transduction currents in isolated vestibular hair cells of the chick. J. Physiol. 359, 189–217. 10.1113/jphysiol.1985.sp015581 2582113 PMC1193371

[B80] Oliver D. He D. Z. Klöcker N. Ludwig J. Schulte U. Waldegger S.. (2001). Intracellular anions as the voltage sensor of prestin, the outer hair cell motor protein. Science 292, 2340–2343. 10.1126/science.1060939 11423665

[B81] Pan B. Geleoc G. S. Asai Y. Horwitz G. C. Kurima K. Ishikawa K.. (2013). TMC1 and TMC2 are components of the mechanotransduction channel in hair cells of the mammalian inner ear. Neuron 79, 504–515. 10.1016/j.neuron.2013.06.019 23871232 PMC3827726

[B82] Pan B. Waguespack J. Schnee M. E. LeBlanc C. Ricci A. J. (2012). Permeation properties of the hair cell mechanotransducer channel provide insight into its molecular structure. J. Neurophysiol. 107, 2408–2420. 10.1152/jn.01178.2011 22323630 PMC3362243

[B83] Peng A. W. Effertz T. Ricci A. J. (2013). Adaptation of mammalian auditory hair cell mechanotransduction is independent of calcium entry. Neuron 80, 960–972. 10.1016/j.neuron.2013.08.025 24267652 PMC4111567

[B84] Peng A. W. Gnanasambandam R. Sachs F. Ricci A. J. (2016). Adaptation independent modulation of auditory hair cell mechanotransduction channel open probability implicates a role for the lipid bilayer. J. Neurosci. 36, 2945–2956. 10.1523/JNEUROSCI.3011-15.2016 26961949 PMC4783497

[B85] Petit M. M. Schoenmakers E. F. Huysmans C. Geurts J. M. Mandahl N. Van de Ven W. J. (1999). LHFP, a novel translocation partner gene of HMGIC in a lipoma, is a member of a new family of LHFP-like genes. Genomics 57, 438–441. 10.1006/geno.1999.5778 10329012

[B86] Pickles J. O. Comis S. D. Osborne M. P. (1984). Cross-links between stereocilia in the guinea pig organ of Corti and their possible relation to sensory transduction. Hear. Res. 15, 103–112. 10.1016/0378-5955(84)90041-8 6436216

[B87] Riazuddin S. Belyantseva I. A. Giese A. P. Lee K. Indzhykulian A. A. Nandamuri S. P.. (2012). Alterations of the CIB2 calcium- and integrin-binding protein cause Usher syndrome type 1J and nonsyndromic deafness DFNB48. Nat. Genet. 44, 1265–1271. 10.1038/ng.2426 23023331 PMC3501259

[B90] Ricci A. J. Crawford A. C. Fettiplace R. (2003). Tonotopic variation in the conductance of the hair cell mechanotransducer channel. Neuron 40, 983–990. 10.1016/s0896-6273(03)00721-9 14659096

[B88] Ricci A. J. Fettiplace R. (1997). The effects of calcium buffering and cyclic AMP on mechano-electrical transduction in turtle auditory hair cells. J. Physiol. 501, 111–124. 10.1111/j.1469-7793.1997.111bo.x 9174998 PMC1159508

[B89] Ricci A. J. Fettiplace R. (1998). Calcium permeation of the turtle hair cell mechanotransducer channel and its relation to the composition of endolymph. J. Physiol. 506, 159–173. 10.1111/j.1469-7793.1998.159bx.x 9481679 PMC2230715

[B91] Ricci A. J. Kennedy H. J. Crawford A. C. Fettiplace R. (2005). The transduction channel filter in auditory hair cells. J. Neurosci. 25, 7831–7839. 10.1523/JNEUROSCI.1127-05.2005 16120785 PMC6725256

[B92] Rybalchenko V. Santos-Sacchi J. (2003). Cl- flux through a non-selective, stretch-sensitive conductance influences the outer hair cell motor of the guinea-pig. J. Physiol. 547, 873–891. 10.1113/jphysiol.2002.036434 12562920 PMC2342734

[B93] Safieddine S. El-Amraoui A. Petit C. (2012). The auditory hair cell ribbon synapse: from assembly to function. Annu. Rev. Neurosci. 35, 509–528. 10.1146/annurev-neuro-061010-113705 22715884

[B94] Scheffer D. I. Shen J. Corey D. P. Chen Z. Y. (2015). Gene expression by mouse inner ear hair cells during development. J. Neurosci. 35, 6366–6380. 10.1523/JNEUROSCI.5126-14.2015 25904789 PMC4405555

[B95] Schwander M. Kachar B. Muller U. (2010). Review series: the cell biology of hearing. J. Cell Biol. 190, 9–20. 10.1083/jcb.201001138 20624897 PMC2911669

[B96] Stepanyan R. Frolenkov G. I. (2009). Fast adaptation and Ca^2+^ sensitivity of the mechanotransducer require myosin-XVa in inner but not outer cochlear hair cells. J. Neurosci. 29, 4023–4034. 10.1523/JNEUROSCI.4566-08.2009 19339598 PMC2702482

[B97] Vreugde S. Erven A. Kros C. J. Marcotti W. Fuchs H. Kurima K.. (2002). Beethoven, a mouse model for dominant, progressive hearing loss DFNA36. Nat. Genet. 30, 257–258. 10.1038/ng848 11850623

[B98] Waguespack J. Salles F. T. Kachar B. Ricci A. J. (2007). Stepwise morphological and functional maturation of mechanotransduction in rat outer hair cells. J. Neurosci. 27, 13890–13902. 10.1523/JNEUROSCI.2159-07.2007 18077701 PMC6673611

[B200] Wang X. Li G. Liu J. Liu J. Xu X. Z. (2016). TMC-1 mediates alkaline sensation in *C. elegans* through nociceptive neurons. Neuron 91, 146–154. 10.1016/j.neuron.2016.05.023 27321925 PMC4938749

[B99] Wang Y. Li J. Yao X. Li W. Du H. Tang M.. (2017). Loss of CIB2 causes profound hearing loss and abolishes mechanoelectrical transduction in mice. Front. Mol. Neurosci. 10:401. 10.3389/fnmol.2017.00401 29255404 PMC5722843

[B102] Wu Z. Grillet N. Zhao B. Cunningham C. Harkins-Perry S. Coste B.. (2017). Mechanosensory hair cells express two molecularly distinct mechanotransduction channels. Nat. Neurosci. 20, 24–33. 10.1038/nn.4449 27893727 PMC5191906

[B101] Wu Z. Müller U. (2016). Molecular identity of the mechanotransduction channel in hair cells: not quiet there yet. J. Neurosci. 36, 10927–10934. 10.1523/JNEUROSCI.1149-16.2016 27798175 PMC5098834

[B100] Wu Y. C. Ricci A. J. Fettiplace R. (1999). Two components of transducer adaptation in auditory hair cells. J. Neurophysiol. 82, 2171–2181. 10.1152/jn.1999.82.5.2171 10561397

[B103] Xiong W. Grillet N. Elledge H. M. Wagner T. F. Zhao B. Johnson K. R.. (2012). TMHS is an integral component of the mechanotransduction machinery of cochlear hair cells. Cell 151, 1283–1295. 10.1016/j.cell.2012.10.041 23217710 PMC3522178

[B104] Yue X. Zhao J. Li X. Fan Y. Duan D. Zhang X.. (2018). TMC proteins modulate egg laying and membrane excitability through a background leak conductance in *C. elegans*. Neuron 97, 571.e5–585.e5. 10.1016/j.neuron.2017.12.041 29395910 PMC7038793

[B106] Zhang Y. V. Aikin T. J. Li Z. Montell C. (2016). The basis of food texture sensation in *Drosophila*. Neuron 91, 863–877. 10.1016/j.neuron.2016.07.013 27478019 PMC4990472

[B105] Zhang L. Gualberto D. G. Guo X. Correa P. Jee C. Garcia L. R. (2015). TMC-1 attenuates *C. elegans* development and sexual behaviour in a chemically defined food environment. Nat. Commun. 6:6345. 10.1038/ncomms7345 25695879

[B107] Zhao B. Wu Z. Grillet N. Yan L. Xiong W. Harkins-Perry S.. (2014). TMIE is an essential component of the mechanotransduction machinery of cochlear hair cells. Neuron 84, 954–967. 10.1016/j.neuron.2014.10.041 25467981 PMC4258123

[B108] Zheng J. Shen W. He D. Z. Long K. B. Madison L. D. Dallos P. (2000). Prestin is the motor protein of cochlear outer hair cells. Nature 405, 149–155. 10.1038/35012009 10821263

